# Knowledge, perceptions, beliefs, and opinions of the employees about GBV: a national online study in South Africa

**DOI:** 10.1186/s12905-023-02704-6

**Published:** 2023-11-02

**Authors:** Corne Davis, Anesu Kuhudzai, Koustuv Dalal

**Affiliations:** 1 Strategic communication, University of Johannesburg, Auckland Park Campus, Johannesburg, South Africa; 2 Division of Public Health Science, School of Health Sciences, Mid Sweden University, Sundsvall, Sweden

**Keywords:** Employee knowledge, Perceptions, Beliefs, Gender-based Violence, GBVPREV questionnaire, South Africa

## Abstract

**Background:**

GBV has been global public health, family, and social problem for several decades as it is expensive for society and the economy. The study was conducted to determine the possible differences in knowledge, perceptions, beliefs, and opinions about GBV, as a whole, across gender and employment sectors in South Africa.

**Methods:**

This was a cross-sectional study using mixed-method design where we used an online survey with two open-ended questions. Perception about GBV Prevention among Employees (GBVPREV) questionnaire consisting of six Sect. (43 questions) was developed and tested. Cronbach’s alpha, Exploratory factor analysis (EFA), including Kaiser-Meyer Olkin Measure of Sampling Adequacy (KMO) and Bartlett’s test of Sphericity, one-way analysis of variance (ANOVA) and Tukey HSD were used. Content analysis was used for analysing qualitative information from two open-ended questions.

**Results:**

Among the 2 270 employees, more than half (68.1%) were females. Males were 677 (29.8%), and members of the LGBTQIAP + community were 32 (1.4%). There were statistically significant differences among males, females, and LGBTQIAP + on employee knowledge of adult experiences, employee knowledge of violence against children, employee perceptions, employee beliefs, and employee opinions and recommendations. The employees believe that all sectors of society should collaborate in addressing GBV in South Africa. They felt that victims should be encouraged to come forward, that society should be less judgemental, that stigma should be addressed and that there should be more empathy for victims.

**Conclusion:**

Most of the respondents, who were female and had tertiary education, were employed in the private sector, and were very aware of the prevalence of GBV in South Africa, agreed that support for both victims and perpetrators must be provided in private sector organizations. Even though it has been acknowledged for decades that gender inequality and GBV are reciprocal drivers, the persistence of both human rights violations will continue if all stakeholders do not collaborate.

**Supplementary Information:**

The online version contains supplementary material available at 10.1186/s12905-023-02704-6.

## Introduction

Gender-based violence (GBV) is term covering a range of violent behaviours, including sexual, physical, psychological, and economic abuse which does not discriminate as it affects all sexual orientations, ages, races, ethnicity, religion, and socioeconomic status [1, 2]. Both victims and perpetrators of GBV can have a combination of all these risk factors including demographic and psychographic characteristics. Often perpetuated through patriarchy, GBV predominantly affects women and girls, severely disrupting women’s rights, a stand-alone barrier to achieving sustainable development goals (SDGs) [3]. The World Health Organization defines *violence* as “the intentional use of physical force or power, threatened or actual, against oneself, another person, or against a group or community, that either result in or has a high likelihood of resulting in injury, death, psychological harm, maldevelopment or deprivation” [4]. Different kinds of GBV disproportionately affect the victims of all socioeconomic statuses worldwide with short and long-term effects on health, relation, society, healthcare systems, judicial systems, and economy [1–5]. Norms relating to the acceptability of GBV have been proven to affect a victim’s likelihood of being abused [5–9]. However, victims from poor socioeconomic status, low educational status, and ethnic minorities are more affected by severe social, economic, physical, and mental health consequences of GBV [10].

GBV has been global public health, family, and social problem for several decades as it is expensive for society and the economy. The world GBV cost estimation was $1.5 trillion [11]. A recent estimation of the economic cost of GBV for South Africa was R36 billion [12]. South Africa has one of the highest worldwide prevalence of GBVs, e.g. female homicide rate is six time higher than the global average [12–14]. The prevalence of physical and Sexual Intimate Partner Violence for a lifetime is 213% and for the last 12 months is 87% 15. Literature indicated that lifetime experience of GBV among women in South Africa ranges between 36 and 77% [14].

There have been increasing demands for GBV prevention, integrating collaborative approaches involving all sectors. However, many policies, publicly funded projects, and programs still need to make significant progress toward GBV control, prevention, and elimination [14]. Literature suggests that different perspectives are needed for GBV prevention [14, 16]. While GBV has initially been addressed primarily from legal and non-government organizational/activist perspectives, it is now also being addressed from public health, governmental, and private sector perspectives. Literature is enriched, focusing on the victims’ perspectives across different socioeconomic status. However, employees of organized sectors, who are supposed to have better socioeconomic statuses at different levels (compared to poor or unorganized sectors), are underexplored for GBV prevention. Literature had concretely advocated that “GBV is the responsibility of stakeholders across all sectors and the importance of the private sector’s participation and intervention, in particular, has to be clearly articulated “ [14]. Many GBV interventions are part of private organizations’ corporate social responsibility (CSR). Employees of private and public organizations include both victims and perpetrators, who require engagement with GBV problems as they are both sensitive and stigmatizing issues. It is necessary to explore GBV perceptions and opinions among employees so that employers can better plan their health and wellbeing programs [12, 14, 16]. However, the literature on assessing employees’ perspectives on GBV is limited. In a country such as South Africa, where GBV statistics are five times higher than the global average, much scientific exploration is warranted [13, 14]. There is also a lack of context-dependent questionnaires querying perceptions of GBV Prevention among Employees (GBVPREV).

The study was conducted to determine the possible differences in knowledge, perceptions, beliefs, and opinions about GBV, across gender and employment sectors in South Africa. A questionnaire querying perception of GBV Prevention among Employees (GBVPREV) was developed and tested here.

## Methods

### Design of the study

This was a cross-sectional study using mixed-method design where we used an online survey with two open-ended questions. An online survey was conducted from July 2021 – May 2022 for ten months. Pre-tested questionnaires were used to develop a survey web-link. Initially, the survey link was shared on LinkedIn. The snowball sampling method, a chain referral mechanism, was used to get as many responses as possible. Snowball sampling in LinkedIn is effective to get employees opinion and feedback, regardless of their position and job title. During the COVID-19 pandemic LinkedIn based survey has emerged as very effective and popular. Due to a lack of previous studies and literature support in the same context, the sample size could not be estimated with any accuracy. This has been supported by the literature [17]. A total of 2875 participants participated in the survey. Among them, 59 participants did not consent to participate in the survey after reading the ethical information. However, all of them filled in the responses. Due to their ethical denial, they were excluded from the study. Also, 546 participants did not complete the survey even though they provided ethical consent. Finally, 2270 participants were included in the study.

### Questionnaire

The questionnaire was developed based on a literature review and experts’ consultations to obtain information regarding employees’ perceptions, opinions, and attitudes towards gender-based violence (GBV) as a problem that affects all stakeholders negatively, including private sector organizations and society at large (supplementary material 1). The questionnaire was pre-tested among a pilot group of 30 respondents. Necessary changes were made, and the ‘consent form’ was added. The Cronbach’s alpha coefficient was adopted to estimate the reliability of the measurement scales. As presented in Table 1, the Cronbach’s alpha value of each construct exceeded the required level of 0.70, supporting the internal consistency and high reliability of the proposed constructs [18].

**Table 1:** Exploratory and Reliability Analysis Results

Dimension	Item	Factor Loadings	Kaiser-Meyer Olkin Measure of Sampling Adequacy (KMO)	Bartlett’s Test of Sphericity	Cronbach’s Alpha Coefficient
Employee Opinions and Recommendations	EO5	0.832	0.958	< 0.001	0.957
	ER2	0.822			
	ER3	0.822			
	EO1	0.810			
	ER4	0.804			
	EO6	0.796			
	ER5	0.794			
	EO2	0.790			
	EO4	0.778			
	EO7	0.754			
	EO3	0.742			
	ER1	0.725			
	ER6	0.653			
	ER7	0.633			
Employee Knowledge of Violence against Children	EK18	0.804			0.837
	EK16	0.716			
	EK15	0.702			
	EK14	0.689			
	EK17	0.651			
	EK13	0.511			
Employee Perceptions	EP3	0.685			0.769
	EP4	0.652			
	EP5	0.651			
	EP2	0.639			
	EP7	0.608			
	EP1	0.570			
	EP6	0.451			
Employee Knowledge of Adult Experiences	EK2	0.782			0.776
	EK3	0.776			
	EK1	0.677			
	EK4	0.589			
Employee Beliefs	EB3	0.805			0.755
	EB2	0.678			
	EB4	0.627			
	EB6	0.479			

The questionnaire Perception about GBV Prevention among Employees (GBVPREV) has six sections consisting of 43 questions, as stated below.

Section A has seven questions focusing on the demographical information of the respondents to enable comparison of responses in terms of gender, age, home language, place of residence, sector of employment, and level of education. Gender has the following five categorical options male, female, LGBTQIAP+, prefer not to say and others. Home language has six options English, Afrikaans, Nguni (isiZulu, isiXhosa, SiSwati, isiNdebele), Sotho (Sepedi, SeSotho, Setswana), Venda/Tsonga and others. Sectors of employment has seven categories to choose from Private sector organizations (profit making organizations), Government sector organizations (funded by the government budget), Non-Government Organizations (not-for-profit organizations), Legal sector organizations, Educational organizations, Public Health organizations and others. South African legal sectors have both public and private organizations. For example, Public legal systems include National prosecuting authority. Private legal system includes private law firms and security firms who work for profit. South African education sectors have both public and private institutions. Public education system provides primary, secondary & tertiary level education funded by the government. Private educational institutions work for profit. Public health organizations include only government funded institutions.

Section B has 18 questions focusing on respondents’ knowledge of the different kinds of GBV identified in existing literature, with responses ranging on a five-point scale from hardly ever in specific socioeconomic sectors to very frequently across all socioeconomic sectors.

All sections C to E have seven questions focusing on employees’ general perceptions of GBV, general beliefs about GBV, and opinions on the role private sector organizations should play in addressing GBV.

Section F (7 questions) focused on recommendations for addressing GBV in private sector organizations in South Africa. All the questions in sections C to F used a five-point Likert scale ranging from strongly disagree to strongly agree.

The questionnaire ended with two open-ended questions for further comments and recommendations.

### Data Analysis

In this study, researchers engaged descriptive statistics and one-way analysis of variance (ANOVA). Descriptive statistics in frequency distributions were used to assess the demographic profile of participants. Descriptive statistics allow the researchers to present the data obtained in a structured, accurate, and summarised manner [19]. One-Way ANOVA was employed to determine the possible differences in knowledge, perceptions, beliefs, and opinions about GBV across gender and employment sectors in South Africa. Where statistically significant differences were noted, multiple comparison tests using the Tukey HSD were performed to investigate where the differences lie. The significance level was assessed at p < 0.05. In this study, the central limit theorem was used to assume that the data was approximately normally distributed since the data set was large (greater than 30). Hence, the parametric tests were employed.

Two open-ended questions were there at the end of the survey. ‘What other comments about GBV would you like to make?’ and second question was ‘What other suggestions do you have as a stakeholder for addressing GBV?’ The open-ended questions were analysed using ‘content analysis’ methodology suggested by Graneheim and Lundman [20]. Computer-based (Atlas-ti software) analyses enabled our meaning unit, condensation, abstraction, coding, categorization and thematising. The trustworthiness of the qualitative analyses was ensured in terms of four key constructs, namely credibility/authenticity, transferability, dependability, and confirmability [20].

### Validity and reliability

Exploratory factor analysis (EFA) was conducted in this study as a construct or factor validity technique. Construct validity refers to determining whether individual items are loaded onto the constructs or factors in the questionnaire [21]. Before performing EFA, two diagnostic measures, Kaiser-Meyer Olkin Measure of Sampling Adequacy (KMO) and Bartlett’s test of Sphericity, were used to assess the factorability of the data. Specifically, the KMO test was utilized to check the sampling adequacy of the data. Bartlett’s Test of Sphericity was used to check if correlations between items were sufficiently strong enough for EFA to be performed. Barlett’s test of Sphericity should reach a statistical significance of less than 0.05 for factor analysis to be suitable [22]. The KMO index value ranges from 0 to 1, with 0.6 considered as the minimum value for exploratory factor analysis to be appropriate [23]. The KMO and Barlett’s test results are presented in Table 1 below. It is evident from Table 1 that the KMO verified the sampling adequacy for the analysis with the KMO value = 0.689 above the threshold value of 0.6 [23]. The Bartlet Test of Sphericity was statistically significant (p-value < 0.05), indicating a solid correlation structure to conduct EFA.

A Principal Components Analysis (PCA) with varimax rotation was performed on 46 items assessing perceptions, opinions, and attitudes toward gender-based violence (GBV). Applying Kaiser’s Guttman rule, five factors were extracted from the factor solution [24]. This rule suggests that only factors with an eigenvalue of 1 and above are retained in the factor solution for further analysis.

Factor 1 was labeled “Employee Opinions and Recommendations” due to high loadings from fourteen items. This factor, with an eigenvalue of 12.079, accounted for 34.511% of the total variance. Factor 2 was interpreted as “Employee Knowledge of Violence against Children.“ This construct, with an eigenvalue of 3.900, explained 11.143% of the total variance. Factor 3 was named “Employee Perceptions.“ This dimension, with an eigenvalue of 1.875, contributed 5.358% of the total variance. Factor 4 was entitled “Employee Knowledge of Adult Experiences.“ This latent variable with an eigenvalue of 1.528 explained 4.365% of the total variance. The last factor was “Employee Beliefs” accountable for high loadings from four items. This component accounted for 3.635% of the total variance. The cumulative variance explained by all five factors is 59.011%, considered very good [25]. Eleven items were deleted from the factor solution due to low factor loadings below 0.4 [26].

IBM Statistical Package for the Social Sciences (SPSS) version 28 was used to generate the descriptive statistics and One-Way ANOVA analysis.

## Results

Among the 2 270 respondents (mean age 37.17 ± s.d.11.37), majority were in the age group of 25–34 years (33.7%), more than two-thirds were females (1 545). Males were 677 (29.8%), and members of the LGBTQIAP + community were 32 (1.4%). The majority of respondents, 1 356 (59.6%), reported working for the private sector; the least was from the public health sector 27 (1.2%). Finally, regarding the highest level of education, most respondents had an honors degree 615 (27.1%), and a few had not matriculated 23 (1.0%). Majority respondents were from Gauteng (66.2%) followed by Western Cape (11.5%), KwaZulu-Natal (8.4%), Eastern Cape (4.1%), Mpumalanga (2.6%), Limpopo (2.4%) Free State (2.1%) and Northern Cape (0.7%). Table 2 shows the profile of respondents according to gender, age, employment sectors, the highest level of education and province of living.

**Table 2:** Demographic Information

Characteristic	Category	n	Percentage
Gender	Male	677	29.8%
	Female	1 545	68.1%
	LGBTQIAP+	32	1.4%
	Prefer not to say	14	0.6%
	Other	2	0.1%
Age group	Up to 24 years	297	13.2%
	25–34 years	756	33.7%
	35–44 years	584	26.0%
	45–54 years	426	19.0%
	55–64 years	153	6.8%
	65 years above	28	1.3%
Employment Sectors	Private sector Organizations	1 356	59.7%
	Government sector Organizations	186	8.2%
	Non-Government Organizations	112	4.9%
	Legal sector Organizations	146	6.4%
	Educational Organizations	331	14.6%
	Public Health Organizations	27	1.2%
	Other	112	4.9%
Highest Level of Education	Have not completed school	23	1.0%
	Matriculated	294	13.0%
	In-Service Training	47	2.1%
	Diploma	350	15.4%
	Degree	609	26.9%
	Honours Degree	615	27.1%
	Masters Degree	288	12.7%
	Doctorate	41	1.8%
Province of living	Mpumalanga	58	2.6%
	Free State	47	2.1%
	Western Cape	261	11.5%
	KwaZulu-Natal	191	8.4%
	Eastern Cape	92	4.1%
	Northern Cape	17	0.7%
	North West	47	2.1%
	Gauteng	1503	66.2%
	Limpopo	54	2.4%

### One Way-ANOVA tests

The one-way ANOVA tests (Table 3) suggest that statistically significant differences existed among males, females, and LGBTQIAP + on employee knowledge of adult experiences, employee knowledge of violence against children, employee perceptions, employee beliefs, and employee opinions and recommendations (p < 0.05). It can also be seen in Table 3 that female employees had more knowledge of adult-related experiences and violence against children than male employees and members of the LGBTQIAP + community. On the other hand, members of the LGBTQIAP + had higher perceptions and beliefs than male and female employees. Finally, female and LGBTQIAP + employees had similar opinions and recommendations in addressing GBV higher than their male counterparts.

**Table 3:** One-Way Analysis Results across gender on perceptions about GBV prevention and intervention

	Males	Females	LGBTQIAP+	
	Mean (SD)	Mean (SD)	Mean (SD)	*p-value*
Employee Knowledge of Adult Experiences	4.39 (0.72)	4.70 (0.52)	4.69 (0.43)	< 0.001
Employee Knowledge of Violence Against Children	3.79 (0.83)	4.19 (0.70)	4.14 (0.76)	< 0.001
Employee Perceptions	3.93 (0.59)	4.25 (0.55)	4.29 (0.64)	< 0.001
Employee Beliefs	4.49 (0.59)	4.63 (0.54)	4.68 (0.67)	< 0.001
Employee Opinions and Recommendations	4.36 (0.64)	4.58 (0.52)	4.58 (0.68)	< 0.001
N	677	1 545	48	

**Pairwise Comparisons**: Post hoc Tests on Employee Knowledge of Adult Experiences revealed significant differences between (Males: M = 4.39, SD = 0.72 vs. Females: M = 4.70, SD = 0.52; p < 0.05) and (Males: M = 4.39, SD = 0.72 vs. LGBTQIAP+: M = 4.69, SD = 0.43; p = 0.002).

Multiple Comparison tests on Employee Knowledge of Violence against Children showed statistically significant differences between (Males: M = 3.79, SD = 0.83 vs. Females: M = 4.19, SD = 0.70; p < 0.05) and (Males: M = 3.79, SD = 0.83 vs. LGBTQIAP+: M = 4.14, SD = 0.76; p = 0.005).

Pairwise Comparison tests on Employee Perceptions suggested that significant differences exist between (Males: M = 3.93, SD = 0.59 vs. Females: M = 4.25, SD = 0.55; p < 0.05) and (Males: M = 3.79, SD = 0.83 vs. LGBTQIAP+: M = 4.29, SD = 0.64; p < 0.05).

Multiple Comparison tests on Employee Beliefs indicated statistically significant differences between (Males: M = 4.49, SD = 0.59 vs. Females: M = 4.63, SD = 0.54; p < 0.05).

Post hoc Tests on Employee Opinions and Recommendations revealed significant differences between (Males: M = 4.36, SD = 0.64 vs. Females: M = 4.58, SD = 0.52; p < 0.05) and (Males: M = 4.36, SD = 0.64 vs. LGBTQIAP+: M = 4.58, SD = 0.68; p = 0.025).

The results in Table 4 indicate no statistically significant differences across the sector in employee knowledge of adult experiences, employee knowledge of violence against children, and employee beliefs, as indicated by the p-values > 0.05 level. Statistically significant differences across the sectors were noted in employee perceptions, opinions, and recommendations, as revealed by the p-values < 0.05 significance level.

**Table 4:** One-Way Analysis Results across sector on perceptions about GBV prevention and intervention

	Private	Government	Non-Government	Legal	Education	Public Health	Other	
	Mean (SD)	Mean (SD)	Mean (SD)	Mean (SD)	Mean (SD)	Mean (SD)	Mean (SD)	*p-value*
Employee Knowledge of Adult Experiences	4.61 (0.59)	4.68 (0.62)	4.50 (0.73)	4.64 (0.64)	4.61 (0.56)	4.57 (0.43)	4.55 (0.74)	0.291
Employee Knowledge of Violence Against Children	4.09 (0.75)	4.12 (0.76)	4.00 (0.92)	3.98 (0.83)	4.05 (0.73)	3.90 (0.69)	4.11 (0.79)	0.364
Employee Perceptions	4.11 (0.57)	4.25 (0.63)	4.28 (0.56)	4.14 (0.58)	4.22 (0.60)	4.10 (0.54)	4.19 (0.54)	0.002
Employee Beliefs	4.57 (0.57)	4.66 (0.47)	4.71 (0.46)	4.59 (0.63)	4.59 (0.59)	4.60 (0.36)	4.58 (0.51)	0.137
Employee Opinions and Recommendations	4.45 (0.60)	4.66 (0.45)	4.65 (0.46)	4.53 (0.55)	4.63 (0.49)	4.57 (0.40)	4.55 (0.53)	< 0.001
N	1 356	186	112	146	331	27	112	

**Pairwise Comparisons**: Post hoc Tests on Employee Perceptions revealed statistically significant differences between (Private: M = 4.11, SD = 0.57 vs. Government: M = 4.25, SD = 0.63; p = 0.04).

Pairwise Comparison tests on Employee Opinions and Recommendations suggested that significant differences exist between (Private: M = 4.45, SD = 0.60 vs. Government: M = 4.66, SD = 0.45; p < 0.05), (Private: M = 4.45, SD = 0.60 vs. Non-Government: M = 4.65, SD = 0.46; p = 0.003) and (Private: M = 4.45, SD = 0.60 vs. Education: M = 4.63, SD = 0.49; p < 0.05).

Since there were significant statistically meaningful differences in employee perceptions, opinions, and recommendations among the various sectors, it is ideal to identify where these differences lie across the various sectors by performing the multiple comparison tests using the Tukey HSD, as stated below.

**Pairwise Comparisons**: Multiple Comparison Tests on Employee Perceptions of GBV revealed significant difference between (Private employees: M = 4.11, SD = 0.57 vs. Government employees: M = 4.25, SD = 0.63; p < 0.05). Multiple Comparison Tests on employee opinions and recommendations by different sector illustrated highly significant results (Private employees: M = 4.45, SD = 0.60 vs. Government employees: M = 4.66 SD = 0.45 p < 0.05; vs. employees from non-government organizations: M = 4.65, SD = 0.46, p = 0.003; vs. Employees from education sector: Education M = 4.63, SD = 0.49, p < 0.05).

There are (Table 5) significant differences in employee knowledge of adult experiences and violence against children among employees with different levels of education. There is no statistically significant difference across the highest level of education on employee perceptions, beliefs, opinions, and recommendations.

**Table 5:** One-Way Analysis Results across highest level of education on perceptions about GBV prevention and intervention

	Below Matric	Matriculated	In-Service Training	Diploma	Degree	Honours Degree	Masters Degree	Doctorate	
	Mean (SD)	Mean (SD)	Mean (SD)	Mean (SD)	Mean (SD)	Mean (SD)	Mean (SD)	Mean (SD)	*p-value*
Employee Knowledge of Adult Experiences	4.38 (0.69)	4.54 (0.68)	4.43 (0.74)	4.61 (0.63)	4.63 (0.58)	4.65 (0.56)	4.62 (0.54)	4.46 (0.81)	0.015
Employee Knowledge of Violence Against Children	3.94 (0.85)	4.15 (0.78)	3.99 (0.92)	4.11 (0.77)	4.06 (0.77)	4.09 (0.74)	3.98 (0.72)	3.80 (0.86)	0.039
Employee Perceptions	4.17 (0.55)	4.12 (0.64)	4.13 (0.61)	4.13 (0.60)	4.16 (0.57)	4.18 (0.53)	4.16 (0.61)	4.14 (0.61)	0.902
Employee Beliefs	4.36 (0.96)	4.54 (0.56)	4.57 (0.53)	4.57 (0.58)	4.62 (0.51)	4.59 (0.58)	4.59 (0.57)	4.68 (0.43)	0.214
Employee Opinions and Recommendations	4.53 (0.58)	4.43 (0.62)	4.42 (0.58)	4.49 (0.59)	4.56 (0.53)	4.52 (0.53)	4.51 (0.60)	4.49 (0.65)	0.075
N	23	294	47	350	609	615	288	41	

### Qualitative results from open-ended questions

The employee’s belief that all sectors of society should collaborate in addressing GBV in South Africa. They felt that victims should be encouraged to come forward, that society should be less judgemental, that stigma should be addressed and that there should be more empathy for victims.

The education and raising awareness are widely supported strategy to deal with GBV. Employees felt that children should be educated about GBV at school, starting at primary school, supported by education in the media and a focus on educating boys and men. The government actions are required to quell the scourge of GBV in South Africa. Employees referred to their views that government was not doing enough, that sentences for perpetrators were too lenient, that there was a lack of trust in the police who were seen to be insensitive and insufficient. Government is not doing enough as GBV perpetrators need harsher punishments. GBV has far-reaching impact. It affects many people, affects productivity and hinders economic growth. The men need to take action, indicating that men need to seek help, and address patriarchy and toxic masculinity. The private sector had to reconceptualise their strategy to address GBV more directly. Employees say financial support is a significant issue. They think that GBV intervention and prevention should be integrated in corporate governance criteria. They feel that more resources are required, that perpetrators require support and that more should be done to achieve gender equality and financial independence for women who remain in abusive relationships because they depend on the perpetrator.

## Discussions

To the best of the authors’ knowledge, this is the first study in Africa focusing on knowledge, perceptions, beliefs, and opinions about GBV among employees from all sectors. A previous European study has tried to assess employees’ social and cultural representations of GBV from a small community [27], but no other study had focused on employees from all sectors at the national level, such as the current study from South Africa. Even though the current study from South Africa was an online survey and the same invitations were sent to the employees from all sectors, public sector employees responded the least (1.2%), and private sector employees responded the highest (59.2%). Respondent’s age distribution shows same pattern as the age distribution of the labor force participation rate in South Africa. Women respondents were more than two-thirds of the total. This indicates how the women employees in South Africa are more eager to express their opinion on GBV. Interestingly, only 1.2% of employees from the public health sector have responded, which could be alarming. The United Nations (UN) and World Health Organizations (WHO) have reiterated that GBV is a significant public health problem and invited all member countries to act for GBV prevention using active public health personnel and strategies [2, 4, 28]. Legal, education and public health sectors were separated in the results. Employees under ‘Legal’ heading work in public judiciary systems such as judge, private law practitioners such as advocates. Also, private security employees were included here. Education consists of employees from both public and private sectors, as both sectors are highly prevalent in South Africa. As public health approach for GBV prevention has been emphasized by all the WHO member countries including South Africa, the current study has tried to get public health workers perspectives [4, 7, 29]. It is therefore interesting to find in the current study that there was such low participation from the public health sector, which could possibly because of desensitization or lack of LinkedIn use by the public health staff. However, further verification and investigation are warranted. Statistically significant differences observed across employment sectors in employee perceptions, opinions, and recommendations, which indicated that employees in different organized sectors have different online access, including social media and/or awareness of GBV. Certainly, education emerged as an important factor for employee knowledge of adult experiences and violence against children. Therefore, policymakers and employers can plan to introduce different awareness and knowledge generation programs on GBV prevention. As education is the most important channel for GBV prevention, employees with better knowledge of GBV prevention can play a better role in the society. Also, compulsory course/ syllabus on GBV prevention at the ground level education system in South Africa could have better impact on knowledge, perceptions, beliefs, and opinions about GBV.

Women employees had more knowledge of adult-related experiences and violence against children than male employees and members of the LGBTQIAP + community. This could be because the women respondents have more personal experience and/or informative knowledge from their peers, families, and neighborhoods [30]. It was reported in South African media in August 2021 that that more than 23 000 teenagers gave birth to babies during covid-19 lockdown, among whom 934 girls were younger than 14 years old [31].

It could be that the women learn GBV as their survival strategies at home and the workplace. Also, literature shows that women, from their girlhood, are socialized to be sincere, supportive, and expressive. Men from their boyhood are socialized to be skilled, competent, independent, and non-disclosing [32]. In this study, employees from the LGBTQIAP + community had stronger perceptions and beliefs about GBV, which could be explained by their experiences and relating to sexual orientation and gender identity. There were also significant differences between the perceptions of employees in different sectors. However, in terms of the variable ‘knowledge’ of adult experiences, violence against children, and their beliefs, there were not significant differences. This could be due to their sexual orientation and gender identity. Gender identity refers to an individual’s sense of who they are, in other words, male, female or other gender. Sexual orientation refers to what gender a person is attracted to within the categories of heterosexual, gay/homosexual, or bisexual. Even though the employees in the study work in different sectors, their gender identity played a more prominent role in their responses.

GBV is also more prevalent for sexual and gender minorities, which might seriously affect their health and wellbeing [33]. It is generally known that intimate partner violence is the most prevalent form of GBV that affect women and girls most, followed by other gendered persons and, to a smaller extent, men.

Education has significantly affected employees’ knowledge of adult experience and violence against children. This has been well explained in literature that shows how education on what GBV encompasses, what GBV interventions have been successful, how and where to find support, and so forth [1–4, 11, 15, 27].

This research is the first online survey where employees from all sectors were asked about GBV in South Africa. This was important because the private sectors employ the majority of people in South Africa and because there has been little evidence of their participation in addressing GBV. Also, the provincial distribution of the respondents is important to notice here. Gauteng is the economic capital and most of the working people are around the area and had highest proportion of survey responses. Western Cape and Kwazulu-Natal have business areas and relatively higher population densities but have 11.5% and 8.4% respondents, respectively. There could have less users of LinkedIn. In the other areas the population densities are thin, and number of employees could be very low. LinkedIn users among the employees those areas could be very sacredly distributed. With the snowball sampling, as the survey started in the Gauteng areas, most of the snowball effect could be around that area. However, this could be our presumption as no specific information is available for further explanations.

The GBVPREV questionnaires were developed based on literature and experts’ consultations, piloting and then validated. The Cronbach’s alpha value of each construct exceeded the required level of 0.70, supporting the internal consistency and high reliability of the proposed constructs, indicating that our questionnaires have added the necessary instrument for the subject.

Categorization of the employment has only seven sectors, finalized by literature review, experts’ comments and pilot testing process. In South Africa, legal sectors and education sectors have both public and private organizations (as stated in method section). Private and public sector employees within these two categories could have been considered. However, as the questionnaire including its categories were developed, modified, validated and confirmed by above mentioned three-stage methods, we should maintain the current employment sector categories, in GBVPREV questionnaire.

Mixed-method study design provide a deeper understanding of the meaning and implications of the result. However, further studies involving more background, risk, and intervention factors, including multivariate studies, are required. Two open-ended questions (‘What other comments about GBV would you like to make?’ and ‘What other suggestions do you have as a stakeholder for addressing GBV?’) were in the survey to complement the quantitative responses. Therefore, more qualitative studies are highly warranted to better understand why there are such differences in knowledge, perceptions, beliefs, and opinions about GBV across the gender continuum and employment sectors in South Africa.

## Conclusion

With most participants in this study being female, employed in the private sector, and representing diverse language groups and geographic locations in South Africa, it is clear that the prevalence of GBV is known among the sample in this study. The knowledge and awareness of the violence experienced by children are alarming. The findings in this study have received significant attention in South African media, and the report has been widely distributed among employers who can take heed of employees’ need for support. Stigma remains the challenge, and the concerted efforts of private sector organizations as critical stakeholders are essential to educate, inform, rehabilitate, and support both victims and perpetrators of GBV. They are well represented in the private sector in South Africa. There is still a significant knowledge gap as GBV is not included sufficiently in business or strategic discourses. There is much room for multi-disciplinary collaboration and a strong call for more academic leadership, participation, and collaboration in working with the private sector for GBV prevention.

### Electronic supplementary material

Below is the link to the electronic supplementary material.

Supplementary Material 1

## Data Availability

The datasets generated and analyzed for this study will be provided at reasonable request to Dr. Corne Davis Email: cdavis@uj.ac.za.
