# Supplementary material for: Knowledge, perceptions, beliefs, and opinions of the employees about GBV: a national online study in South Africa
Source: BMC Womens Health. 2023 Nov 2;23:565. doi: 10.1186/s12905-023-02704-6 (PMC10621235; doi:10.1186/s12905-023-02704-6)
Supplement: Supplementary file 1 — Supplementary Material 1 [file 12905_2023_2704_MOESM1_ESM.docx]

**PERCEPTION ABOUT GBV PREVENTION AMONG EMPLOYEES (GBVPREV) QUESTIONNAIRE**

**SCREENING QUESTIONS**

By giving your consent, you agree to partake in this survey. Your responses will remain anonymous and will be kept confidential. No personal identifying information is required to complete the survey. Please know that you may withdraw at any point.

1. Do you give your consent to participate in this study?

| No, I do not give my consent to participate in this study. | 0 |
| --- | --- |
| Yes, I give my consent to participate in this survey and understand that I can withdraw at any time. | 1 |

***** If your answer is yes, please complete the remainder of the questionnaire.***

**SECTION A: BIOGRAPHICAL DETAILS**

2. What is your gender?

| Male | 1 |
| --- | --- |
| Female | 2 |
| LGBTQIAP+ | 3 |
| Prefer not to say | 4 |
| Other, please specify | 5 |

3. What is your age?

| Answer |  |
| --- | --- |

4. What is your home language?

| English | 1 |
| --- | --- |
| Afrikaans | 2 |
| Nguni (isiZulu, isiXhosa, SiSwati, isiNdebele) | 3 |
| Sotho (Sepedi, SeSotho, Setswana) | 4 |
| Venda/Tsonga | 5 |
| Other, please specify | 6 |

5. In which province in South Africa do you live?

| Mpumalanga | 1 |
| --- | --- |
| Free State | 2 |
| Western Cape | 3 |
| KwaZulu-Natal | 4 |
| Eastern Cape | 5 |
| Northern Cape | 6 |
| North West | 7 |
| Gauteng | 8 |
| Limpopo | 9 |

6. In which sector do you work?

| Private | 1 |
| --- | --- |
| Government | 2 |
| Non-government | 3 |
| Legal | 4 |
| Education | 5 |
| Public health | 6 |
| Other | 7 |

7. If in the **private sector**, in which industry do you work?

| Not Applicable (If not in the private sector) | 1 |
| --- | --- |
| Energy | 2 |
| Materials | 3 |
| Industrials | 4 |
| Consumer Discretionary | 5 |
| Consumer Staples | 6 |
| Health Care | 7 |
| Financials | 8 |
| Information Technology | 9 |
| Not sure | 10 |
| Other, specify………………………………………….. | 11 |

8. What is your highest level of education?

| Have not completed school | 1 |
| --- | --- |
| Matriculated | 2 |
| In-service training | 3 |
| Diploma | 4 |
| Degree | 5 |
| Honours degree | 6 |
| Masters degree | 7 |
| Doctorate | 8 |

**___________________________________________________________________**

**SECTION B: EMPLOYEE KNOWLEDGE OF GBV IN SOUTH AFRICA**

To your knowledge, how frequently do the following kinds of gender-based violence occur in South Africa? 1 = hardly ever occurs, 2 = occurs sometimes in specific socio-economic sectors, 3 = not sure, 4 = occurs sometimes across all socio-economic sectors, 5 = occurs frequently across all socio-economic sectors

|  | | Hardly ever in specific socio-economic sectors → Very frequently across all socio-economic sectors | | | | |
| --- | --- | --- | --- | --- | --- | --- |
| EK1 | Femicide (Killing of women by their intimate partners) |  |  |  |  |  |
| EK2 | Intimate relationship partner violence | 1 | 2 | 3 | 4 | 5 |
| EK3 | Domestic violence (non-sexual violence in the home) | 1 | 2 | 3 | 4 | 5 |
| EK4 | Rape of women by strangers | 1 | 2 | 3 | 4 | 5 |
| EK5 | Male rape (rape of men by other men) | 1 | 2 | 3 | 4 | 5 |
| EK6 | Male rape by women | 1 | 2 | 3 | 4 | 5 |
| EK7 | Corrective rape (rape of lesbian women by men to “fix” their orientation | 1 | 2 | 3 | 4 | 5 |
| EK8 | Blessers (mostly married men dating students for sex in exchange for money and gifts) | 1 | 2 | 3 | 4 | 5 |
| EK9 | Sexual harassment at work (paying unwanted sexual to a person in the workplace) | 1 | 2 | 3 | 4 | 5 |
| EK10 | Sexual harassment in public places (paying unwanted sexual attention to a person in public places) | 1 | 2 | 3 | 4 | 5 |
| EK11 | Educators/lecturers offering marks to students in exchange for sex | 1 | 2 | 3 | 4 | 5 |
| EK12 | Students offering sex to educators/lecturers in exchange for marks | 1 | 2 | 3 | 4 | 5 |
| EK13 | Physical violence against children | 1 | 2 | 3 | 4 | 5 |
| EK14 | Human trafficking (kidnapping mostly children for sexual exploitation) | 1 | 2 | 3 | 4 | 5 |
| EK15 | Ukuthwala (forced marriage of female children) | 1 | 2 | 3 | 4 | 5 |
| EK16 | Online child pornography (posting/watching online material of children being sexually exploited or raped) | 1 | 2 | 3 | 4 | 5 |
| EK17 | Child rape of boys or girls | 1 | 2 | 3 | 4 | 5 |
| EK18 | Child prostitution (enabling the sexual exploitation of minor children in exchange for money or goods) | 1 | 2 | 3 | 4 | 5 |

**SECTION C: EMPLOYEE PERCEPTIONS OF GBV IN SOUTH AFRICA**

On a scale of 1 to 5 where 1= Strongly Disagree, 2= Disagree, 3= Neither Disagree nor Agree, 4= Agree, 5= Strongly Agree, indicate the extent to which you agree with each of the following statements.

|  | | Strongly Disagree → Strongly Agree | | | | |
| --- | --- | --- | --- | --- | --- | --- |
| EP1 | I think financial dependence is one of the reasons why victims stay in abusive relationships | 1 | 2 | 3 | 4 | 5 |
| EP2 | I think family pressures and fear of stigma prevent victims from seeking help | 1 | 2 | 3 | 4 | 5 |
| EP3 | I think GBV plays a big role in women’s career progress or lack thereof | 1 | 2 | 3 | 4 | 5 |
| EP4 | I think gender inequality is a big driver of GBV | 1 | 2 | 3 | 4 | 5 |
| EP5 | I think fear of losing one’s job contributes to the silence about Intimate Partner Violence, Violence Against Children and Domestic Violence | 1 | 2 | 3 | 4 | 5 |
| EP6 | I think there are people in my organisation that may be victims of Intimate Partner Violence or Domestic Violence | 1 | 2 | 3 | 4 | 5 |
| EP7 | I think GBV has a big impact on workplace productivity | 1 | 2 | 3 | 4 | 5 |

**SECTION D: EMPLOYEE BELIEFS ABOUT GBV**

On a scale of 1 to 5 where 1 = Strongly Disagree, 2= Disagree, 3= Neither Disagree nor Agree, 4= Agree and 5= Strongly Agree, indicate the extent to which you agree with each of the following statements.

|  |  | Strongly Disagree → Strongly Agree | | | | |
| --- | --- | --- | --- | --- | --- | --- |
| EB1 | I believe that GBV is a personal matter that should not be addressed at work | 1 | 2 | 3 | 4 | 5 |
| EB2 | I believe that GBV affects work and support should be available at the workplace | 1 | 2 | 3 | 4 | 5 |
| EB3 | I believe that victims should be able to speak out without being discriminated against | 1 | 2 | 3 | 4 | 5 |
| EB4 | I believe that people who judge victims are part of the GBV problem | 1 | 2 | 3 | 4 | 5 |
| EB5 | I believe that women can be very judgmental of women who experience GBV | 1 | 2 | 3 | 4 | 5 |
| EB6 | I believe that GBV can be reduced if all stakeholders work together | 1 | 2 | 3 | 4 | 5 |
| EB7 | I believe that both victims and perpetrators of GBV need support | 1 | 2 | 3 | 4 | 5 |

**SECTION E: EMPLOYEE OPINIONS ON ORGANISATIONAL RESPONSIBILITY**

On a scale of 1 to 5 where 1 = Strongly Disagree, 2= Disagree, 3= Neither Disagree nor Agree, 4= Agree and 5= Strongly Agree, indicate the extent to which you agree with each of the following statements.

|  |  | Strongly Disagree → Strongly Agree | | | | |
| --- | --- | --- | --- | --- | --- | --- |
| EO1 | In my opinion private sector organisations should become involved in addressing GBV as a social responsibility concern | 1 | 2 | 3 | 4 | 5 |
| EO2 | In my opinion private sector organisations should provide employees with information about the prevalence of GBV in South Africa. | 1 | 2 | 3 | 4 | 5 |
| EO3 | In my opinion private sector organisations should acknowledge that employees may be victims and/or perpetrators of GBV | 1 | 2 | 3 | 4 | 5 |
| EO4 | In my opinion private sector organisations should focus on all forms of GBV and not just sexual harassment in their policies | 1 | 2 | 3 | 4 | 5 |
| EO5 | In my opinion private sector organisations should develop GBV support initiatives to educate employees | 1 | 2 | 3 | 4 | 5 |
| EO6 | In my opinion private sector organisations should include GBV in employee wellbeing programmes | 1 | 2 | 3 | 4 | 5 |
| EO7 | In my opinion private sector organisations should include details of their GBV support initiatives in their annual integrated reports | 1 | 2 | 3 | 4 | 5 |

**SECTION F: EMPLOYEE RECOMMENDATIONS FOR ADDRESSING GBV**

On a scale of 1 to 5 where 1 = Strongly Disagree, 2= Disagree, 3= Neither Disagree nor Agree, 4= Agree and 5= Strongly Agree, indicate the extent to which you agree with each of the following statements.

|  |  | Strongly Disagree → Strongly Agree | | | | |
| --- | --- | --- | --- | --- | --- | --- |
| ER1 | I recommend that private sector organisations collaborate with government in addressing GBV | 1 | 2 | 3 | 4 | 5 |
| ER2 | I recommend that private sector organisations work with NGOs to address GBV | 1 | 2 | 3 | 4 | 5 |
| ER3 | I recommend that private sector organisations collaborate with each other in addressing GBV | 1 | 2 | 3 | 4 | 5 |
| ER4 | I recommend that private sector organisations should collaborate with universities to conduct research on GBV | 1 | 2 | 3 | 4 | 5 |
| ER5 | I recommend that private sector organisations participate in developing GBV support as an occupational health and safety issue | 1 | 2 | 3 | 4 | 5 |
| ER6 | I recommend that private sector organisations change cultures of toxic masculinity, patriarchy and male supremacy | 1 | 2 | 3 | 4 | 5 |
| ER7 | I recommend that private sector organisations do more to ensure equal opportunities and salaries for women | 1 | 2 | 3 | 4 | 5 |

**SECTION G: EMPLOYEE COMMENTS**

1. What other comments about GBV would you like to make?
2. What other suggestions do you have as a stakeholder for addressing GBV?

**Thank you for taking the time to complete this survey!**
